# Imported malaria in a non-endemic area: the experience of the university of Campinas hospital in the Brazilian Southeast

**DOI:** 10.1186/1475-2875-13-280

**Published:** 2014-07-22

**Authors:** João C K Dos-Santos, Rodrigo N Angerami, Catarina M S Castiñeiras, Stefanie C P Lopes, Letusa Albrecht, Márcia T Garcia, Carlos E Levy, Maria L Moretti, Marcus V G Lacerda, Fabio T M Costa

**Affiliations:** 1 Departamento de Genética, Evolução e Bioagentes, Instituo de Biologia, Universidade Estadual de Campinas (UNICAMP), Campinas, SP, Brazil; 2 Faculdade de Ciências Médicas, UNICAMP, Campinas, SP, Brazil; 3 Seção de Epidemiologia Hospitalar, Hospital das Clínicas, UNICAMP, Campinas, SP, Brazil; 4 Departamento de Clínica Médica, Faculdade de Ciências Médicas, UNICAMP, Campinas, SP, Brazil; 5 Departamento de Patologia Clínica, UNICAMP, Campinas, SP, Brazil; 6 Fundação de Medicina Tropical Dr. Heitor Vieira Dourado, Manaus, AM, Brazil; 7 Universidade do Estado do Amazonas, Manaus, AM, Brazil

**Keywords:** Malaria, Non-endemic area, Brazil, *Plasmodium vivax*, *Plasmodium falciparum*, Severity, Epidemiology

## Abstract

**Background:**

Although malaria in Brazil almost exclusively occurs within the boundaries of the Amazon Region, some concerns are raised regarding imported malaria to non-endemic areas of the country, notably increased incidence of complications due to delayed diagnoses. However, although imported malaria in Brazil represents a major health problem, only a few studies have addressed this subject.

**Methods:**

A retrospective case series is presented in which 263 medical charts were analysed to investigate the clinical and epidemiological characterization of malaria cases that were diagnosed and treated at Hospital & Clinics, State University of Campinas between 1998 and 2011.

**Results:**

Amongst all medical charts analysed, 224 patients had a parasitological confirmed diagnosis of malaria. *Plasmodium vivax* and *Plasmodium falciparum* were responsible for 67% and 30% of the infections, respectively. The majority of patients were male (83%) of a productive age (median, 37 years old). Importantly, severe complications did not differ significantly between *P. vivax* (14 cases, 9%) and *P. falciparum* (7 cases, 10%) infections.

**Conclusions:**

Severe malaria cases were frequent among imported cases in Brazil outside of the Amazon area. The findings reinforce the idea that *P. vivax* infections in Brazil are not benign, regardless the endemicity of the area studied. Moreover, as the hospital is located in a privileged site, it could be used for future studies of malaria relapses and primaquine resistance mechanisms. Finally, based on the volume of cases treated and the secondary complications, referral malaria services are needed in the non-endemic areas of Brazil for a rapid and efficient and treatment.

## Background

Despite intense global efforts to control malaria, non-immune and semi-immune travellers living in non-endemic areas remain a highly vulnerable group for malaria infection, especially due to delayed diagnosis and increased risks of complications. Therefore, although the clinical outlook for malaria in previously healthy adults living in endemic areas tends to be mild, the absence of immunity and delayed or misdiagnosis (up to 40% [1]) may predispose infected persons to more severe presentations in imported cases of disease. Indeed, the chances of death are 80 to 100 times higher for malaria cases diagnosed outside of endemic areas [1-3].

Approximately 60% of malaria cases in the Americas occur in Brazil [4], and these almost exclusively occur in the Amazon Region. Today, most cases are diagnosed and treated at the same place where infection was acquired within 48 hours of the onset of symptoms, according to the Brazilian Ministry of Health. However, some regions of the country outside of the Amazon Region still receive patients presenting with malaria, either from the Amazon Region or from other countries, mainly Latin American and African nations. Although malaria is a major health problem in Brazil, with 177,783 cases registered in the Amazon Region in 2013 (more than 99% of Brazilian cases occur in the Amazon Region), few studies have analysed the epidemiology and major complications triggered by this infection outside of endemic areas.

The Hospital & Clinics of the State University of Campinas (HC-UNICAMP) is one of the fourteen-referral centers for the diagnosis and treatment of malaria in the Brazilian State of São Paulo. The region was formerly endemic for malaria, with a great number of autochthonous cases in the early decades of the 20th century [5]. In the 1960s, the Malaria Eradication Campaign led to a sharp drop in the number of autochthonous cases and, from 1980 to 2000, only one autochthonous case was reported in the Campinas region (6) [6]. Here, a retrospective case series is presented that analyses 263 medical charts for the clinical and epidemiological characteristics of malaria cases treated at HC-UNICAMP from January 1998 to April 2011.

## Methods

### Ethical approval

The informed consent waiver was approved by the Research Ethics Committee of the University of Campinas (number 1283/2011).

### Study design and subject enrollment

The study was designed as a retrospective case series and took place at the HC-UNICAMP. Hospital & Clinics, State University of Campinas (S 22° 49′, W 47° 03′, Campinas, Brazil) is a tertiary hospital with 409 beds; 471,338 medical consultations and emergency treatments were performed in 2012. The hospital has a coverage area of more than 100 municipalities in the areas surrounding Campinas, which includes a population of 5,000,000 in these municipalities [7].

Patients seen at HC-UNICAMP who received a confirmed diagnosis of malaria that was reported by its Hospital Epidemiology Division (NVE-UNICAMP) were selected. Diagnoses had been confirmed based on analysis of thick and thin blood films (TBF) that were examined by trained experienced staff. Patients were enrolled in this study, regardless of the species of *Plasmodium* infection identified, from January 1998 to April 2011. PCR was not routinely performed to confirm the *Plasmodium* species that caused an infection; however a senior microscopist systematically performed quality control. Only patients with accessible medical charts were enrolled. A standard questionnaire was used to register data obtained from the medical charts, such as demographic profile, time of clinical disease, referred previous malarial infections, travel history, peripheral blood parasitaemia, clinical and laboratory profiles, physical examination and the prescribed anti-malarial regimen.

Malaria patients at HC-UNICAMP are treated following the Brazilian guidelines for the treatment of malaria (Brazilian Ministry of Health, 2010) [8]. Patients were categorized as severe or non-severe according to 2010 World Health Organization (WHO) criteria [9]. WHO does not have a guideline to define malaria severity in infections by *P. vivax*. Since its absence, Lacerda et al. [10] argued that the WHO criteria used for *P. falciparum* infection fits malaria severity found in *P. vivax* infection. Therefore, the same criteria for both species were used. Because some patients were treated for malaria at the HC-UNICAMP for two or more episodes of malaria, only the first confirmed case registered on the medical chart was considered in the analysis. Patients were divided into residents (n = 56) and travellers (n = 87) based on the time that they stayed in the endemic area; a stay of more than 12 months was characterized as residence. Differences for which *P* < 0.05 were considered to be statistically significant.

### Relapses

After treatment, TBF were performed to verify cure. Blood films were checked at days 15, 45, 75 and 105 after treatment for patients with *P. vivax* malaria. Relapses were defined as the cases in which, after treatment and a negative TBF, subjects presented a new TBF with a positive result more than 28 days after starting anti-malarial treatment. Moreover, a case was only considered to be a relapse if the patient did not return to an endemic area during the follow-up period.

### Drug regimens

Drug regimens followed Brazilian guidelines for the treatment of malaria (Brazilian Ministry of Health, 2010) [8]. Patients with uncomplicated *P. vivax* malaria received non-supervised chloroquine for three days (four pills on day 1 and three pills on both days 2 and 3) and non-supervised primaquine (15 mg daily for 14 days). In cases of true relapse, the dose of primaquine was increased to 22.5 mg/day for 14 days; if there was a second relapse, the dose was increased to 30.0 mg/day for 14 days. Patients with uncomplicated *P. falciparum* malaria received a single oral mefloquine dose at admission (prior to 2007) or artesunate plus mefloquine p.o. for three days (2007–2011). Intramuscular artemether was given to 18 patients with severe disease. There was no information available on chemoprophylaxis.

### Statistical analyses

Fisher’s exact test was used for categorical data. Student’s *t*-test was used to compare groups with normally distributed data, and data sets with non-normal distributions were compared using the Mann–Whitney test. GraphPad Prism 5® and Stata 9® software were used for these analysis.

## Results

### General findings

**Figure 1:**
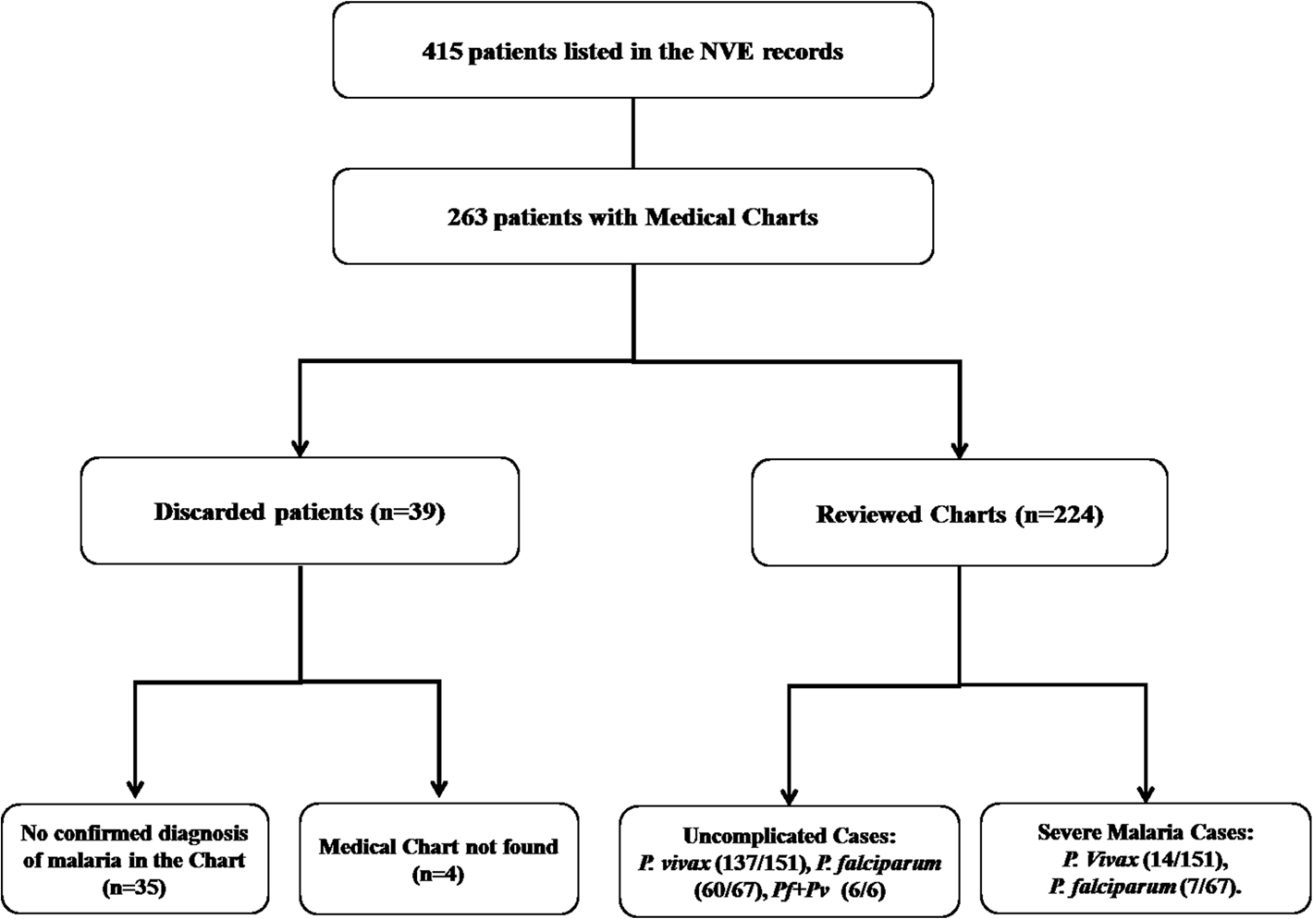
**A flow diagram of medical chart selection.** There were 415 names retrieved from the Center for Epidemiological Surveillance at UNICAMP. Of these, 263 were of patients with medical charts, but 35 of them had no confirmed diagnosis of malaria by TBF and 4 charts were not found, so only 224 charts were reviewed.

**Figure 2:**
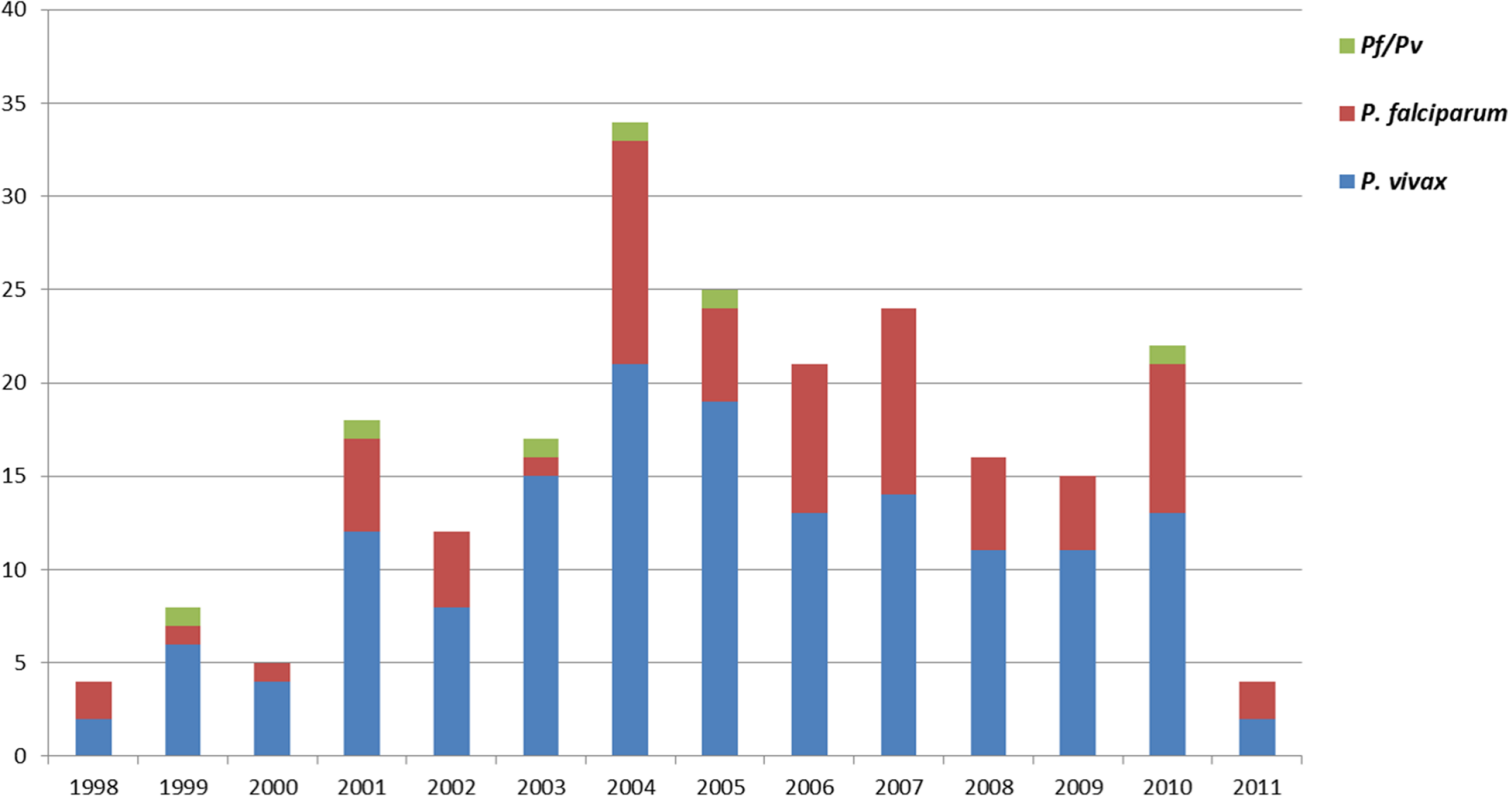
**Distribution of cases by *Plasmodium *species and year.** The distribution of cases by year is shown. No association with the total number of Brazilian cases or any seasonality in the cases was identified. The 2011 cases were recorded until the month of April (n = 224).

Amongst a list of 415 subjects obtained from the NVE in 2011, only 263 patients had charts, of which four charts could not be retrieved and 35 cases were not confirmed as malarial infections after microscopic diagnosis. Therefore, clinical and epidemiological data were only retrieved from 224 patients (Figure 1). *Plasmodium vivax* malaria accounted for the majority of the cases (n = 151; 67%), while *P. falciparum* was identified in 67 patients (30%) and mixed infection (*P. vivax* + *P. falciparum*) was found in only six patients. No cases of infection by other *Plasmodium* species were reported. The distribution of cases per species and year is shown in Figure 2. The majority of infected people were men (n = 186; 83%) for both *P. vivax* and *P. falciparum* infections (Tables 1 and 2). Eleven patients (7%) who were at least 30 years old had diabetes—a proportion very close to that of the Brazilian population of the same age group—and 17 patients (21%) who were at least 45 years old had hypertension. Three patients (1%) had AIDS, and one of these cases had progressed to severe disease due to *P. vivax*.

**Table 1:** Demographic profile of malaria patients at HC-UNICAMP in the period 1998-2011

	**Infectious species**					
**Variable**	***P. falciparum***		***P. vivax***		***Pf/Pv***	
	**Freq.**	**(%)**	**Freq**	**(%)**	**Freq.**	**(%)**
N	68/224	(30)	150/224	(67)	6/224	(3)
Gender:						
Male	59/67	(88)^a^	124/151	(82)	3/6	(50)^a^
Female	8/68	(12)	27/150	(18)	3/6	(50)
Age:						
Mean ± SD	35	±13	40	±16	26	±18
Median [p25-p75]	35	[26-45]^a^	39	[28-52]^a^	19	[15-41]
Ethnicity:						
White	45/65	(69)	117/148	(79)	5/6	(83)
Mixed	12/65	(18)	27/148	(18)	1/6	(17)
Black	8/68	(12)	4/148	(3)	0/6	(0)

^a^*P* < 0,05.

**Table 2:** Clinical profile of malaria patients at HC-UNICAMP in the period 1998-2011

**Variable**	**Infectious species**				***P *value**
	***P. falciparum***		***P. vivax***		
Days of symptoms prior to diagnosis	N = 53		N = 100		
Median [IQR]	4	[3-7]	6	[4-9]	0,0326
Hospitalization (%)	35/67	(52)	30/151	(20)	<0,0001
Days of hospitalization	N = 35		N = 29		
Median [IQR]	4	[2-6]	3	[2-4]	0,1701
Days since return from endemic area	N = 40		N = 59		
Median [IQR]	10	[5-14]	15	[8-25]	0,0078
Months in endemic area*	N = 29		N = 57		
Median [IQR]	1	[0,6-4,5]	1	[0,5-2,5]	0,2136
Referred fever (%)	61/67	(91)	125/151	(83)	0,1465
Referred shivers (%)	29/67	(43)	73/151	(48)	0,5569
Referred headache (%)	45/67	(67)	80/151	(53)	0,0550
Referred myalgia (%)	33/67	(49)	69/151	(46)	0,6607
Arthralgia (%)	10/11	(91)	22/32	(69)	0,2370
Vomiting (%)	25/36	(69)	24/44	(55)	0,2488
Sweating (%)	10/14	(71)	17/28	(61)	0,7337
Tachypnea (%)	9/18	(50)	5/12	(42)	0,7220
Jaundice (%)	22/34	(65)	19/46	(41)	0,0448
Splenomegaly (%)	22/43	(51)	20/58	(34)	0,1060

*Only patients considered to be travellers (<12 months in endemic area).

The possible place of infection could be retrieved in 205 cases. Of these, 168 infections (82%) took place in Brazil and 37 cases were from other countries, those were called ‘international’ cases, representing 18% of the total. The leading foreign country regarding the number of cases was Angola (13 cases, 6.3%), followed by Haiti and Guiana (3 cases each), French Guiana, Ghana, Congo and Mozambique (2 cases each), and Venezuela, Cape Verde, Guinea Bissau, Equatorial Guinea, Cameroon, Egypt, South Africa and Papua New Guinea (1 case each).

### Malaria clinical complications and severity

Severe malaria was defined according to the WHO criteria [9,10]. Among 21 patients (9%) who presented with severe malaria, there were 14 cases of *P. vivax* infections, seven cases of *P. falciparum* infections Therefore, 9% of the *P. vivax* infections and 10% of the *P. falciparum* infections evolved into severe disease; these proportions were not significantly different (*P* > 0.05). Notably, no patient with severe disease was a resident of the probable area of infection, as five months was the longest stay.

Six of the 224 patients (3%) presented with spontaneous bleeding of the nose and/or gums. All of these patients had *P. vivax* malaria, but no significant difference for this sign between *P. falciparum* and *P. vivax* was found. Four of these six patients presented thrombocytopaenia (platelet count <150,000/mm^3^), which is not a criterion for severe malaria according to WHO guidelines [9].

From the 41 patients with available urine test data, eight subjects had haemoglobinuria. These cases included six *P. vivax* infections (4% of the total *P. vivax* malaria cases) and two *P. falciparum* infections (3% of the total *P. falciparum* malaria cases). Among the four patients who presented with type 1 respiratory failure (pO_2_ < 60 mmHg and pCO_2_ < 35 mmHg) [11], three had *P. falciparum* malaria and one had *P. vivax* malaria.

Hyperbilirubinaemia (total bilirubin >3.0 mg/dL) is considered to be a marker of severe disease only if it is associated with another indicator of organ dysfunction [12]. Following this criterion, five patients presenting with hyperbilirubinaemia and renal failure were considered to be patients with severe disease. For renal failure, it was considered a creatinine level >1.5 mg/dl [13,14]. Of note, this value does not correspond to the WHO criterion for renal failure in severe malaria, and the patients were considered to have severe malaria for the concomitant presence of an altered serum creatinine and serum bilirubin. No patient met the WHO criteria for severe anemia, cerebral malaria, circulatory collapse, hypoglycaemia, acidosis or hyperlactataemia.

### Malaria relapses

There were 19 (12.6%) cases of true relapses amongst patients infected with *P. vivax* infections. For these relapsing episodes, the drug regimen was either maintained or the primaquine dose was increased. Among patients with relapses, four presented no symptoms during the relapse, four required hospitalization and 14 presented with fever. Thrombocytopaenia and anaemia were common findings, occurring in 69% and 60% of patients with relapses, respectively. Most of the relapses resolved after the first retreatment, but five patients (26%) experienced more than one relapse.

### Laboratory admission findings

Thrombocytopaenia (platelet count <150,000/mm^3^) [15] was the most common finding in laboratory tests at the time of diagnosis, affecting 128 of 150 patients (85%). This proportion did not vary significantly between the groups with severe or uncomplicated disease or between *Plasmodium* species. Twenty-eight patients had severe thrombocytopaenia (platelet count <50,000/mm^3^) [16]. Of those, 14 were patients with *P. vivax* malaria (15%) and 14 were patients with *P. falciparum* malaria (27%). The difference between these proportions was not significantly different (Table 3).

**Table 3:** Laboratory admission values of malaria patients at HC-UNICAMP during 1998-2011

**Variable**	**Infectious species**		***P *value**
	***P. falciparum***	***P. vivax***	
Platelets (150-450 × 10^3^/ml)	N = 53	N = 93	0,974
	83 [46,5-122]	81 [53–109,5]	
Haemoglobin (13–17 mg/dl)	N = 55	N = 94	0,507
	13,2 [12,2-14,5]	13,7 [12,6-14,6]	
Haematocrit (42-52%)	N = 41	N = 75	0,806
	40 [37-42]	40 [36-43]	
Erythrocytes (4.5-6.1 × 10^6^/ml)	N = 35	N = 75	0,687
	4,43 [4,06-4,76]	4,51 [4,11-5,01]	
Leukocytes (4-10 × 10^3^/ml)	N = 53	N = 91	0,219
	5,4 [4,0-6,5]	5,4 [4,3-6,8]	
AST (≤37 U/L)	N = 43	N = 61	0,029
	40 [27-71]	32 [20,5-46]	
ALT (≤40 U/L)	N = 42	N = 60	0,088
	42,5 [24,8-97,2]	31,5 [19,5-61,8]	
GGT (≤40 U/L)	N = 21	N = 23	0,204
	61 [33-200]	58 [23-75]	
Total Bilirubin (≤1.0 mg/dl)	N = 35	N = 48	0,210
	1,60 [0,97-4,98]	1,18 [0,95-2,18]	
PT (INR) (≤1.25)	N = 28	N = 36	0,015
	1,24 [1,1,35]	1,15 [1,06-1,24]	
PTT (R) (≤1.30)	N = 28	N = 36	0,401
	1,20 [1,1,33]	1,14 [1,01-1,32]	
LDH (240–479 U/L)	N = 11	N = 12	0,013
	751 [661-838]	464 [397-552]	
Creatinine (0.7-1.3 mg/dl)	N = 45	N = 64	0,647
	1,07 [0,78-1,22]	0,98 [0,88-1,15]	

N refers to the absolute number of patients with the respective laboratory test information available.

Overall, 52/152 patients (34%) were found to be anaemic (Hb < 11 mg/dL for females and Hb < 13 mg/dL for males) [17]. When comparing patients with severe disease to those with non-severe disease, 57% (16/28) of patients in the severe group were anemic compared to 29% (36/124) of patients in the non-severe group. The white blood cell (WBC) count was not different amongst the different groups of patients and a small proportion of patients (17%, 25 of 146 patients) were leukopenic.

Laboratory test results were only different between patients with *P. falciparum* or *P. vivax* malaria for prothrombin time (PT), lactate dehydrogenate (LDH) and aspartate aminotransferase (AST), were slightly elevated only for LDH and AST in *P. falciparum* infections (Table 4). As the sample sizes were small for LDH, a biomarker that was not part of routine evaluations of malaria cases, this difference might be biased. No further investigations were carried out to investigate the difference in AST levels.

**Table 4:** Proportion of clinical signs among severe malaria cases met at HC-UNICAMP during 1998-2011

			**Infecting species**			
**Parameter**	**Overall**		***P. falciparum***		***P. vivax***	
	**Freq**	**(%)**	**Freq**	**(%)**	**Freq**	**(%)**
N	21	(100)	7	(100)	14	(100)
Bleeding	6/21	(29)	0/7	(0)	6/14	(43)
Haemoglobinuria	8/21	(38)	2/7	(29)	6/14	(43)
Hyperbilirrubinaemia + Renal Impairment	6/21	(29)	3/7	(43)	2/14	(14)
Respiratory Failure	4/21	(19)	3/7	(43)	1/14	(7)

There was no difference in the frequency of clinical signs between the infecting species, as calculated with Fisher’s Exact Test.

As expected, a significant decrease in haemoglobin, haematocrit and red blood cell levels in the first days after the onset of treatment was detected (*P* < 0.0001). A significant reduction in WBC was also found, but both median values were within the reference range.

## Discussion

The proportion among of Plasmodium species that caused infection in the present study cohort was different from what is observed in Brazil as a whole (where *P. vivax* accounts for nearly 85% of cases), but was very similar to what has been previously reported for this region [6,7]. A higher incidence of *P. vivax* infections, compared to *P. falciparum* infections, is different from what is observed in Europe and the USA, where most imported cases come from Africa [18-20], but is similar to what is observed in Australia, where most cases come from Asia [16,21]. Notably, although the peak of cases detected in our study in 2004 does not directly mirror the profile of Brazil, where the highest incidence of malaria cases took place in 2005; however, 2004 was still marked by one of the highest number of cases [22]. Otherwise, no special event was found that would fully explain this pattern.

The high frequency of ‘international’ cases derived from Angola (34%) probably reflects the fact that both Brazil and Angola are former Portuguese colonies. The relationship of imported cases in European countries with their former African colonies is a well-known phenomenon [23]. Nonetheless, in contrast to cases included in European studies [24,25], the number of cases associated with visiting friends and relatives in endemic countries was very small in the group of ‘international’ cases. Business travellers and recent African immigrants made up the majority of patients infected in former Portuguese colonies in our study. The lack of patients who had been infected in African countries during travels to visit friends and relatives may indicate that the relationships between two former colonies or a former colony and colonizer differ.

The higher proportion of infected men, a common finding among imported malaria studies [26,27], resulted from the occurrence of most infections when patients travelled to endemic areas, especially for work-related reasons. This result could indicate that either men are employed in jobs associated with the greatest risk of infection or men still more often occupy the position of family provider [28]. This is exemplified by the observation that amongst 111 patients with registered occupations, 21 (19%) were male truck drivers, an occupation at risk of infection during trips to endemic areas. Of some interest was the finding that all cases originating in Haiti (3 cases) affected Brazilian soldiers who were on humanitarian deployments for the UN and were also men of working age.

There was no significant difference in the time elapsed between the onset of symptoms and the initiation of treatment when cases of *P. falciparum* and *P. vivax* were compared. The finding that there was no greater time interval between the onset of symptoms and treatment for cases of complicated *P. vivax* or *P. falciparum* malaria is of critical importance, as it suggests that a longer disease duration and increased parasitaemia are not sufficient to yield complications of *P. vivax* malaria, and the development of severe disease might also depend on other factors intrinsic to the parasite species [29].

Of particular interest is the finding that no patients with severe malaria were residents of endemic areas, which supports the concept of greater vulnerability of non-immune patients, or those with waning immunity, to the development of severe presentations of the disease [1]. This information is of paramount importance, as it serves as a warning to health professionals and systems located far away from endemic areas to be alert for patients presenting with signs of malaria, especially *P. vivax* malaria, which is still perceived to be a benign disease [4,30]. One patient developed respiratory failure due to complicated *P. vivax* malaria, a finding previously reported in other Brazilian studies [31,32]. Six patients with *P. vivax* malaria presented with haemoglobinuria (4% of *P. vivax* cases), a rate of incidence similar to what has been observed previously in a multicenter study of endemic areas [33], but the proportions may vary [34] and previous studies on this topic focused on *P. falciparum* malaria.

Thrombocytopaenia was a common finding that affected 85% of patients analysed, a proportion comparable to other studies of imported malaria [16,35]. This finding reinforces the idea that thrombocytopenia in patients returning from areas endemic for malaria should alert a physician to the possibility of this disease. The prevalence of severe thrombocytopaenia (platelet count <50,000/mm^3^) [16] was also relatively high, presenting in 12.5% of patients. The lack of data correlating platelet levels and bleeding does not prove the absence of an association, as some instances of minor bleeding may have not been registered in the medical charts. Anaemia was found in 34% of patients upon admission, a value close to what has been reported in a previous study on imported malaria in the Canary Islands [20], in which the majority of cases were caused by *P. falciparum*. From the beginning of treatment of patients with anti-malarials, there was a reduction in haemoglobin, haematocrit and red blood cells, as previously reported [36]. This pattern was observed both in patients with *P. falciparum* and *P. vivax* malaria, and anaemia worsened both in patients that received chloroquine and primaquine or mefloquine and artesunate.

Notably, from 2001 to 2011, there were 2391 cases of malaria in São Paulo, with 28 deaths caused by malaria in this state, a lethality rate of 1.1% [37,38]. This lethality rate is more than 30 times greater than the rate in the Amazon area, according to official statistics [37]. Nevertheless, no deaths were observed at the HC-UNICAMP in this study. According to national statistics, there were three deaths in the region of Campinas during the study period, resulting in a lethality rate that was similar to that of the State of São Paulo. Although the lethality of the cases in Brazil that occurred outside of the Amazon Region in our study differs from previous studies [2,3], in which an 80 to 100 times higher lethality rate was reported, the findings in this study still support the notion that cases in non-endemic areas of Brazil are associated with an augmented risk of complications.

Analysing the incidence of relapsing cases along with associated factors is important for preventing such events. Relapses are dangerous because of their morbidity and risk of reintroducing malaria to areas where mosquito vectors are still present [39]. The occurrence of 12.6% *P. vivax* relapses that we found is lower that what was previously observed in other studies of imported malaria in Brazil [40,41], but the lack of monitoring for a cure after a first bout of treatment and distinct study methods might account for this difference. The adequacy of the primaquine dose for the weight of a patient in the HC-UNICAMP was only adopted after the study period and this may contribute to a lower incidence of relapses in the subsequent years. It is also relevant to highlight that as a reference center for malaria control in a non-endemic area, the University of Campinas Hospital is in a privileged location to study the mechanisms of malaria relapse and the development of primaquine resistance in the future.

There was no information about chemoprophylaxis for malaria in the medical charts. Thus, it was impossible to assess different disease severities or the presence of relapses based on this variable. The absence of such information might result from the recommendation by the Ministry of Health that chemoprophylaxis is only advised for persons travelling to areas with a high incidence of *P. falciparum* infection, which does not include most areas of Brazil, but it is important to highlight that prevention does not solely comprise chemoprophylaxis [42]. It is important to highlight that current travel advisory services in Campinas are restricted to the UNICAMP academic community and to medical consultancy in private companies.

### Study limitations

Because this study took place in a tertiary hospital, there may have been some bias in the selection of patients, as subclinical or mild disease may have not compelled infected individuals seek out healthcare. Retrospective case series studies tend to face gaps in the register of information and the record of symptoms is somewhat biased because denied symptoms have a greater chance of not being registered in the medical chart than referred ones. Furthermore, it was not possible to exclude, from the medical charts, the presence of concomitant dengue fever in all of the cases, and this coinfection could explain cases of bleeding. Nevertheless, it was not possible to fully assess the presence of comorbities in all patients enrolled in the study.

The lack of a more sensitive method for malaria diagnosis in this study, especially PCR, makes it impossible to rule out the presence of mixed infection in the cases of severe *vivax* malaria. Nonetheless, TBF are systematically reviewed and a lower incidence of *P. falciparum* infection makes coinfection of *Plasmodium* species less likely.

## Conclusions

This study has gathered much information about malaria in a region of Brazil outside of the Amazon, a subject that has received little attention by researchers. Despite the peculiarities of Brazilian culture, a concordance of the social aspects of imported malaria among the cases studied and of other studies of imported malaria in Latin America was observed. Based on the clinical and laboratory profiles of imported malaria cases in a non-endemic area of Brazil, infection with *P. vivax* was found to be responsible for severe cases. This finding reinforces the idea that *P. vivax* infections in Brazil are not benign and establishes a framework for further research on the topic. Indeed, more studies are needed on malaria in non-endemic areas in Brazil, especially with a prospective design and adequate exclusion of comorbidities and coinfection. Finally, considering the study area, the volume of cases treated and the possibility of secondary complications, the importance of referral malaria services that offer rapid and efficient diagnosis and treatment in Brazil is urgently apparent. There is also academic and scientific value in the study of malaria in non-endemic areas, because it is only in these areas that cases of relapses due to *P. vivax* can be studied without the interference of potential reinfections.

## Competing interests

The authors declare that they have no competing interests.

## Authors’ contributions

JCKD collected the charts, performed and analyzed the data and wrote the first version of the manuscript. RNA, CMSC, SCPL, LA, MTA, CEL and MLM helped in data analysis and in the design of the study. MVGL and FTMC designed the study, helped in data analysis and wrote the final version of the manuscript.
